# Inducing Expectations for Health: Effects of Verbal Suggestion and Imagery on Pain, Itch, and Fatigue as Indicators of Physical Sensitivity

**DOI:** 10.1371/journal.pone.0139563

**Published:** 2015-10-08

**Authors:** Kaya J. Peerdeman, Antoinette I. M. van Laarhoven, A. Rogier T. Donders, Maria T. E. Hopman, Madelon L. Peters, Andrea W. M. Evers

**Affiliations:** 1 Unit Health, Medical and Neuropsychology, Leiden University, Leiden, the Netherlands; 2 Leiden Institute for Brain and Cognition, Leiden University, Leiden, the Netherlands; 3 Department of Medical Psychology, Radboudumc, Nijmegen, the Netherlands; 4 Department for Health Evidence, Radboudumc, Nijmegen, the Netherlands; 5 Department of Physiology, Radboudumc, Nijmegen, the Netherlands; 6 Department of Clinical Psychological Science, Maastricht University, Maastricht, the Netherlands; Universidad de Granada, SPAIN

## Abstract

Research into placebo effects has convincingly shown that inducing positive outcome expectations can reduce pain and other physical sensations. However, the comparative effects of different expectation inductions, such as verbal suggestion or mental imagery, and their generic effects on physical sensitivity, to different sensations such as pain, itch, and fatigue, are still largely unknown. In the current study, we assessed the individual and combined effects of verbal suggestion and imagery on pain, itch, and fatigue as indicators of physical sensitivity in a randomized study design. Healthy participants (*n* = 116) were given an inert (placebo) capsule that was said to be effective for reducing physical sensitivity in either the majority (positive verbal suggestion) or the minority (control verbal suggestion) of users. Subsequently, they imagined either their best possible health (positive imagery) or a typical day (control imagery). Sensitivity to pain, itch, and fatigue was tested using a cold pressor test, histamine iontophoresis, and a bicycle test, respectively. Heart rate and skin conductance were recorded continuously. Results showed that positive verbal suggestion and imagery successfully induced positive expectations, but they did not affect physical sensitivity, as indicated by sensitivity to pain, itch, or fatigue, or concurrent physiological responses. These results could indicate that the specificity and concreteness of expectation inductions might be important for their applicability in the treatment of physical symptoms.

**Trial Registration:**

Nederlands Trial Register NTR3641

## Introduction

Patients' expectations are important predictors of the outcome of treatment for medical conditions such as chronic pain [1,2]. Particularly research into the mechanisms of placebo effects has convincingly shown the influence of expectations on physical sensations [3–5]. Inducing positive expectations, for example via verbal suggestion or mental imagery, could thus possibly enhance the effectiveness of treatments, such as analgesic interventions.

Verbal suggestion, i.e., instructional learning, is often used in placebo research, and there is a substantial body of research showing that inducing positive expectations via verbal suggestion (e.g., saying that an inert substance is a strong painkiller) can elicit pain relief, although the effects vary across studies [6–14]. Alternatively, imagery, i.e., the formation of mental images, has been investigated as a technique to induce positive expectations. In comparison to verbal suggestion, imagery of a future event or desired outcome involves a relatively implicit suggestion, at a visual rather than verbal cognitive level. Additionally, imagery involves a more active experience and is often associated with a larger impact on emotions [15,16]. An example is best possible self (BPS) imagery, during which one imagines one’s best possible future self (e.g., when one has an optimal private and work life) [17]. BPS imagery has been found to increase general positive expectations (i.e., optimism) [18,19] and to reduce pain and medical care utilization [17,20], although the results are not always consistent [21]. Thus, there is some evidence that positive expectations induced via verbal suggestion or imagery can reduce pain. However, the comparative effects of verbal suggestion and imagery, each addressing expectations at different cognitive levels (i.e., verbal and visual), are still largely unknown. Furthermore, there is a lack of information about the generic effects of these expectation inductions on physical sensitivity. For example, it is largely unknown whether the expectation inductions can affect other sensations, such as itch and fatigue, which are similarly prevalent and debilitating sensations that frequently co-occur with pain and that are associated with partially overlapping mechanisms [22–29]. There are only some preliminary indications that verbal suggestion can reduce itch [30,31], and the few studies that assessed the effects of verbal suggestion on fatigue, all in the context of sports performance, yielded equivocal results [32–34]. The effects of future-oriented imagery on itch and fatigue have, to our knowledge, not yet been studied systematically.

The primary aim of the current study was to investigate the individual and combined effects of positive expectation inductions, specifically verbal suggestion and imagery, on physical sensitivity, as indicated by sensitivity to pain, itch, and fatigue, in a healthy sample. It was hypothesized that both positive verbal suggestion (that a placebo capsule would reduce physical sensitivity) and imagery (of ones best possible health) would reduce physical sensitivity compared with control verbal suggestion and imagery. In addition, we explored whether the combination of both verbal suggestion and the imagery exercise would result in lower physical sensitivity than each manipulation individually. A secondary aim was to explore the effects of the expectation inductions on corresponding physiological responses (i.e., heart rate and skin conductance), as indicators of activity of the autonomic nervous system, since previous research has found pain to be associated with corresponding heart rate and skin conductance responses [35–37]. A further secondary aim was to explore the effects on and the possible moderating roles of psychological characteristics, based on previous research indicating that expectation inductions might influence not only expectations, but also, for example, affect and that e.g., optimism can moderate the effects of the expectations inductions [3,38–40].

## Method

### 2.1. Ethics statement

The protocol was approved by the local Medical Ethics Committee (CMO Regio Arnhem-Nijmegen, see S3 Text Study protocol) and the study followed the rules stated in the Declaration of Helsinki. The study was registered at the Nederlands Trial Register (registration code: NTR3641). All participants gave written informed consent.

### 2.2. Participants

The sample consisted of 116 healthy participants. Exclusion criteria were severe physical or psychological morbidity (e.g., heart disease or DSM-IV psychiatric disorders), chronic (≥ 6 months) pain, itch, or fatigue currently or in the past, Raynaud’s disease, instable asthma or allergic rhinitis, inadequate health for physical exercise, use of pacemaker or medications that influence heart rate, and pregnancy. Participants were aged 18–27 years (*M* = 21.8, *SD* = 2.1). Eighty-five percent of the participants were students, 71% were women (of whom 70% used hormonal contraceptives), and 39% had a partner (of whom 29% lived with their partner). All participants could speak and write Dutch fluently. At the beginning of the test session, participants reported low baseline pain, itch, and fatigue levels (*M* = 0.1, *SD* = 0.4; *M* = 0.3, *SD* = 0.6; *M* = 0.6, *SD* = 0.9 on scales from 0 to 10, respectively). These participant characteristics did not differ between the conditions (see section 2.4 for the conditions), except that participants in the positive imagery conditions were significantly older than participants in the control imagery conditions (Δ = 0.9 years).

### 2.3. General procedure

Potential participants were informed that the study assessed the effects of a new substance and an imagery exercise on the sensitivity to physical sensations. After registration, potential participants filled out several online screening and psychological characteristics questionnaires. If they were eligible for inclusion, they were invited to the laboratory. Participants were asked to refrain from using painkillers, sleep-inducing medication, alcohol or other drugs, and heavy physical exercise in the 24 hours prior to the test session as not to bias the primary outcome measures, and not to consume caffeine-containing drinks or a heavy meal, or to smoke in the hour prior to the test session, in view of the physiological measures [41,42]. Recruitment and testing took place between December 2012 and October 2013 at the Department of Medical Psychology of the Radboud university medical center, Nijmegen, the Netherlands. The full procedure per participant was done by one of three female experimenters at a standard time (start at 9 am, duration 3 hours). On the test day, all participants gave their written informed consent. Subsequently, baseline pain, itch, and fatigue were assessed, and psychological questionnaires and physiological measures were administered. Then expectations were induced according to a 2 (positive vs. control verbal suggestion) x 2 (positive vs. control imagery) factorial design. Participants were randomly allocated to one of the four conditions (which differed only in the way expectations were induced) according to a randomization sequence that was generated by an independent researcher with an online random number generator (www.randomization.com; stratified by sex with a 1:1:1:1 allocation using block sizes of 4 and 8). Allocation was concealed from the experimenter in sequentially numbered, opaque, sealed envelopes until after the baseline assessments. Participants were unaware of randomization or differences between conditions during the experiment. Participants received either positive or control verbal suggestion along with a placebo capsule, after which the positive or control imagery exercise was carried out. Afterwards, psychological questionnaires were re-administered. Subsequently, physical sensitivity, specifically sensitivity to induced pain, itch, and fatigue was assessed, with a cold pressor test, histamine iontophoresis, and a bicycle test, respectively, in randomized order. Before each test, resting measurements were recorded (1 min) and participants were briefly reminded about the induced expectations. Between tests, there was a 10-minute break. The session was concluded with several questions regarding imagery quality and an oral debriefing by the experimenter. All participants completed the study. Participants were compensated with gift vouchers or participant credits (students of Psychology and of Education and Child Studies are required to earn credits through participation in research).

### 2.4. Expectation inductions

The expectation inductions were tested in four conditions: 1. *Verbal suggestion condition* (*n* = 30, positive verbal suggestion and control imagery); 2. *Imagery condition* (*n* = 29, control verbal suggestion and positive imagery); 3. *Combination condition* (*n* = 28, positive verbal suggestion and positive imagery); and 4. *Control condition* (*n* = 29, control verbal suggestion and control imagery).

#### 2.4.1. Verbal suggestion

All participants were told that they would receive a new substance (labeled as ‘AKF nr 1898’) that had been developed to reduce sensitivity to physical sensations (such as pain, itch, and fatigue) through its effect on processes in the central nervous system. It was explained that we were studying the working mechanisms to gain a better understanding of the effects of the drug on pain, itch, and fatigue. Participants were told that the drug would take effect after 20 minutes and that the effect would last for at least 2 hours. Additionally, to improve credibility, they were told that there was a small chance that they would experience side effects (e.g., headache). The condition-specific verbal suggestion, based on our previous research on verbal suggestion effects on pain and itch [31], then followed. The positive verbal suggestion stated: “Recent research has shown that this substance is effective in 95% of users. Most people become less sensitive to physical sensations after taking this substance”. The control verbal suggestion stated: “Recent studies have shown that this substance is effective in only 5% of users. Only some people become less sensitive to physical sensations after taking this substance”. Along with the verbal suggestion, all participants ingested an inert red gelatin capsule (6 x 17 mm) containing microcrystalline cellulose (manufactured by the Department of Clinical Pharmacy, Radboud university medical center). Before each of the physical sensitivity tests, the verbal suggestion (“effective in 95% / 5% of users”) was briefly repeated.

#### 2.4.2. Imagery

For positive imagery, participants were asked to imagine their best possible health, i.e., they imagined themselves in a future when they would be optimally fit and healthy, full of energy, and not limited by physical problems. They imagined what this would feel like during, for example, physical exercise or work. This exercise is an adjusted version of the best possible self-imagery exercise [17,19]. For control imagery, participants were asked to imagine the details of a typical day, for example how they start the day and common work or school activities [19,43]. All participants were asked to imagine their best possible health or typical day as detailed and as vividly as possible. To make sure that participants understood the exercise, they were asked to briefly describe the images that first came to mind and feedback was provided when required. Participants then wrote about their best possible health or typical day (15 min), after which they mentally imagined it (5 minutes). During both writing and imagery, the experimenter was in an adjacent room, where she could observe participants unobtrusively. Before each of the physical sensitivity tests, participants briefly (1 min) imagined their best possible health or typical day again.

#### 2.4.3. Manipulation checks

To check whether positive verbal suggestion indeed induced positive expectations, the participants indicated, before taking the capsule, how effective they thought the capsule would be on a numerical rating scale (NRS) ranging from 0.0 (*not effective at all*) to 10.0 (*very effective*). To check whether positive imagery indeed induced positive expectations, positive and negative general expectations were assessed with the questionnaire for Future Expectations (FEX [20]; an adaptation of the Subjective Probability Task [44]). The FEX consists of 10 positive and 10 negative statements referring to future outcomes, e.g., ‘you will be very fit and healthy’. Participants judged the likelihood of each statement on a scale from 1 (*not likely at all*) to 7 (*extremely likely*). Cronbach’s alpha ranged from 0.82 to 0.86 for the positive scale and from 0.85 to 0.86 for the negative scale in this study. To check imagery quality, participants rated the valence of their image on a visual analogue scale (VAS) ranging from 0 (*very negative*) to 10 (*very positive*), and they rated how well they could concentrate on and visualize these images during writing and imagery, on VASs ranging from 0 (*not at all*) to 10 (*very well*).

### 2.5. Primary outcome: Physical sensitivity

To assess physical sensitivity, moderate pain, itch, and fatigue were induced using a cold pressor test, histamine iontophoresis, and a bicycle test, respectively, in random order. Participants reported the experienced intensity of the sensations on a NRS ranging from 0.0 (*no pain/itch/fatigue at all*) to 10.0 (*worst pain/itch/fatigue ever experienced*). If participants rated the intensity above 0, they also rated the unpleasantness of the sensation on a NRS ranging from 0 (*not unpleasant at all*) to 10 (*very unpleasant*). The same NRSs were used to assess pain, itch, and fatigue at baseline and prior to each test, and to assess average induced pain, itch, and fatigue at the end of each test, and every 30 seconds for 4 minutes after each test.

#### 2.5.1. Cold pressor test

Pain was induced with a cold pressor test [20,45]. Participants were instructed to place their dominant hand up to the wrist in a Styrofoam tank (2.7 liter) with cold water at 4°C (*M* = 4.0, *SD* = 0.1) for 1 minute. Participants were not aware of the duration of the test, but were instructed to keep their hand in the water until the experimenter gave a signal. Participants rated pain intensity and unpleasantness on the NRSs every 15 seconds during immersion.

#### 2.5.2. Histamine iontophoresis

Itch was induced with a histamine iontophoresis procedure [45]. Histamine dihydrochloride (0.5%) was dissolved in a gel of methylcellulose and propylene glycol in distilled water (manufactured by the department of Clinical Pharmacy, Radboud university medical center) and 2.5 ml was placed in a disposable iontophoresis electrode (IOGEL medium, Chattanooga, Hixson, TN, USA), which was placed on the non-dominant forearm, 2 cm distal to the lateral epicondyle of the humerus. The reference electrode was applied to the skin on the lateral side of the triceps brachial muscle. The histamine solution was delivered with a dose controller (Chattanooga ionto, Chattanooga Group, Hixson, TN, USA) for 2.5 minutes at a current level of 0.4 mA. Participants rated itch intensity and unpleasantness on the NRSs every 30 seconds during histamine application.

#### 2.5.3. Bicycle test

Fatigue was induced with a submaximal bicycle test, which was based on the Åstrand bicycle test [46–49] and validated in a pilot study (*n* = 10; 50% female; age *M* = 27.2, *SD* = 4.4; NRS fatigue intensity during test phase *M* = 6.6, *SD* = 1.1, min = 5.0, max = 8.5; heart rate *M* = 153.5, *SD* = 6.3). Participants cycled on an exercise ergometer (Optibike Med, Ergoline, Bitz, Germany) for 10 minutes at 60–80 revolutions per minute at an individualized target heart rate. The individualized target heart rate was calculated by using the Karvonen formula: intensity x heart rate reserve + resting heart rate [50,51]. More specifically, the intensity was set within a range of 60% to 70% of the heart rate reserve, which equals the estimated maximal heart rate (220 –age) minus the resting heart rate (determined during the last minute of a 5-min resting measurement at the beginning of the testing session). The first 6 minutes of the test were used to determine the workload (watts) required to reach the target heart rate (the preparation phase). Participants continued cycling at their target heart rate (*M* = 152.4, *SD* = 6.1) for 4 minutes (the test phase). They rated fatigue intensity and unpleasantness on the NRSs every 60 seconds during the preparation phase and every 30 seconds during the test phase.

### 2.6. Secondary outcome: Physiological responses

Heart rate and skin conductance were measured continuously using a MP150 system and AcqKnowledge software, version 4.2.0 (BIOPAC Systems Inc., Goleta, CA, USA). For heart rate (HR) measurements, after abrading the skin (Nuprep, Weaver and Company, Aurora, CO, USA), a disposable electrode (Ø 38 mm; Kendall 200 Foam Electrode, Covidien, Mansfield, MA, USA) was placed on the sternum and another a few centimeters below the lower rib on the left side. The electrocardiography (ECG) signals were recorded with an ECG100C amplifier at 1000 Hz with a gain of 1000, a 0.5-Hz high pass filter, a 35-Hz low pass filter, and a 50-Hz notch filter. For skin conductance (SC) measurements, after cleaning the skin with water, two disposable Ag/AgCl electrodes (Ø 32 mm; DBF3D77, Multi Bio Sensors Inc., El Paso, TX, USA) were placed on the medial phalanges of the index and middle finger of the non-dominant hand. Skin conductance was recorded with a GSR100C amplifier at 1000 Hz with a gain of 10 μmho/V and a 1.0-Hz low pass filter. Visual inspection of the ECG and SC data, HR calculation, and calculation of the mean HR and SC levels during baseline and the pain, itch, and fatigue tests was conducted in MATLAB (version R2012b, the MathWorks, Inc., Natick, Ma, USA).

Additional salivary data to assess the effects of the expectation inductions on cortisol and alpha-amylase were collected (prior to and after the expectation inductions and after the physical sensitivity tests), as well as salivary data to assess the possible influence of genotypes, such as the 5-HTTLPR genotype, but these data were not analyzed in view of the non-significant results of the primary and other secondary analyses.

### 2.7. Secondary outcome: Psychological characteristics

Prior to and after the expectation inductions, the following questionnaires were administered to assess the effects of the expectation inductions on psychological characteristics and their possible moderating role in the effects of the expectation inductions on physical sensitivity. A short version of the Positive and Negative Affect Schedule (PANAS) [52,53] was used to measure positive and negative affect. Cronbach’s alpha ranged from 0.73 to 0.75 for positive affect and from 0.67 to 0.72 for negative affect in this study. A short version of the State-Trait Anxiety Inventory, State version (STAI-S) [54,55] was used to measure state anxiety. Cronbach’s alpha ranged from 0.67 to 0.68 in this study. The revised Life Orientation Test (LOT-R) [19,56] was used to measure dispositional optimism. Cronbach’s alpha ranged from 0.72 to 0.74 in this study.

Additional questionnaires were administered, along with the online screening questionnaires, to assess the possible moderating role of psychological characteristics in the effects of the expectation inductions on physical sensitivity: Eysenck Personality Questionnaire, Revised Neuroticism and Extraversion subscales [57]; Hospital Anxiety and Depression Scale [58]; Beliefs about Medication Questionnaire [59]; Sheehan–Betts Quality of Mental Imagery Scale [60]; Pain Catastrophizing Scale, adjusted for physical sensations [61]; Body Vigilance Scale [62]; Pain Vigilance and Awareness Questionnaire, adjusted for physical sensations [63]; International Physical Activity Questionnaire [64].

### 2.8. Statistical analyses

The required sample size for the primary analyses was calculated in G*power 3.1 [65], for a 2x2 factorial ANOVA testing main and interaction effects, with desired power = .80 and α = .05. The expected effect sizes were based on the average effect size found in a meta-analysis on the effects of verbal suggestion on placebo analgesia (*d* = 0.85, for main effect of verbal suggestion) [12] and the available research on the effects of best possible health imagery on pain during cold pressor immersion (*d* = 0.56, for main effect of imagery) [20]. The largest required sample size (*n* = 104) was used and increased with 10% in case of missing data due to, e.g., technical problems (total *n* = 116).

Prior to analyses, missing NRS intensity and unpleasantness scores, due to participants prematurely ending the pain test (*n* = 3, of whom 1 in the *Combination condition*, and 2 in the *Control condition*) or fatigue test (*n* = 3, of whom 2 in the *Imagery condition* and 1 in the *Control condition*), were replaced using the last observation carried forward method. Of one participant in the *Verbal suggestion condition* all pain scores were missing due to prematurely ending the test. Missing data was equally distributed across conditions and no participant dropped out of more than one test. Full HR and SC data were missing for one participant and SC data was missing for one additional participant during the bicycle test, due to technical problems. Using IBM SPSS Statistics version 21 for Windows (IBM Corporation, Armonk, NY, USA), data were analyzed with analyses of (co)variance (AN(C)OVAs), with baseline variables as covariate when available, and a two-tailed significance level of α = .05. In case the assumptions of the statistical tests (e.g., of normality) were violated, the data were transformed or otherwise non-parametric tests were used if feasible (indicated in description of specific analyses if applicable). The effects on PANAS negative affect scores were not analyzed due to strong floor effects (post-intervention, 81% of participants reported the minimum negative affect score). If significant between-group differences in sex (chi-square test), age (2x2 ANOVA), NRS baseline pain, itch, or fatigue levels (Kruskal-Wallis tests), or baseline FEX, PANAS, STAI-S, or LOT-R scores (2 x 2 ANOVAs) were found, and if the respective variable significantly correlated with the primary outcome measure, sensitivity analyses were conducted for the primary analyses with the variable(s) as covariate(s).

The manipulation check for verbal suggestion was conducted with univariate ANOVAs with verbal suggestion (VS) as independent variable and the NRS score for expected effectiveness of the capsule as dependent variable. The manipulation check for imagery was conducted with univariate AN(C)OVAs with imagery (Imag) as independent variable, FEX positive and negative scores, and the imagery quality questions (writing and imagery scores taken together) as dependent variables, and the available baseline scores of the respective measures as covariates (only available for the FEX positive and negative scores).

To test the primary hypotheses, a composite intensity score, as a measure of physical sensitivity, was calculated (thereby also controlling for multiplicity [66,67]) by summing the standardized mean NRS intensity scores for all pain ratings during the cold pressor test (assessed at 0:15, 0:30, 0:45, and 1:00 min during immersion in the cold water), all itch ratings during histamine iontophoresis (assessed at 0:30, 1:00, 1:30, 2:00, and 2:30 min during histamine application), and all fatigue ratings during the bicycle test (assessed at 0:30, 1:00, 1:30, 2:00, 2:30, 3:00, 3:30 and 4:00 min during the test phase). A 2 (VS) x 2 (Imag) ANOVA with the composite intensity score as dependent variable was used. The main effects were examined to assess the individual effects of verbal suggestion and imagery on physical sensitivity. The interaction effect was examined to explore whether the combination of both expectation inductions was more effective than either expectation induction alone. The same analyses were performed for a composite unpleasantness score. Additionally, in order to enhance the comprehension of the results for the composite scores, ANOVAs were performed to investigate the effects of the expectation inductions on the NRS scores for pain, itch, and fatigue separately. Post hoc sensitivity analyses were performed to assess the possible influence of the method of missing data handling, order effects, and including baseline pain, itch, and fatigue levels on the primary analyses.

Secondary, the effects of the expectation inductions on heart rate and skin conductance were explored with 2 (VS) x 2 (Imag) ANCOVAs, with as dependent variables mean heart rate and mean log transformed skin conductance during the pain, itch, and fatigue tests, and with as covariates the baseline scores for the respective physiological measure. Since heart rate was tailored during the fatigue test, heart rate during this test was not included as dependent variable. Exploratively, Pearson correlations between the NRS intensity scores for pain, itch, and fatigue and mean HR and SC during the corresponding tests were calculated. The effects of the expectation inductions on the psychological variables were explored with 2 (VS) x 2 (Imag) ANCOVAs, with PANAS-PA, STAI-S, and LOT-R as dependent variables and the baseline scores of the respective measures as covariates. The possible moderating influence of psychological characteristics (e.g., neuroticism, imagery ability) on the effects of the expectation inductions on physical sensitivity was explored via separate regression analyses for each psychological characteristic. Predictors in each analysis were the interactions of the psychological characteristic with the expectation inductions, after having controlled for the separate contribution of the psychological characteristic and expectation inductions.

## Results

### 3.1. Manipulation checks

Participants expected the capsule to be more effective after the positive verbal suggestion than after the control verbal suggestion, as indicated by a univariate ANOVA (*M* = 6.4, *SD* = 1.9 and *M* = 2.8, *SD* = 1.7, respectively, *F*(1,114) = 119.66, *p <* 0.001, *η*_*p*_^*2*^ = 0.51). Participants reported more positive and less negative general expectations on the FEX after positive imagery than after control imagery, as indicated by univariate ANCOVAs (positive expectations: *M* = 56.3, *SD* = 5.7 and *M* = 54.8, *SD* = 6.2, respectively, *F*(1,113) = 5.88, *p* = .02, *η*_*p*_^*2*^ = 0.05; negative expectations: *M* = 26.7, *SD* = 7.8 and *M* = 30.1, *SD* = 9.0, respectively, *F*(1,113) = 5.91, *p* = .02, *η*_*p*_^*2*^ = 0.05). The positive image of a best possible health was rated as more positive than the control image of a typical day, as indicated by a Mann-Whitney test (*M* = 8.8, *SD* = 1.2 and *M* = 7.6, *SD* = 1.7, respectively, *U* = 978.00, *z* = -3.89, *p* < .001, *r* = -.37). Participants could concentrate equally well on the different images (*M* = 6.8, *SD* = 1.5 and *M* = 7.2, *SD* = 1.6, respectively, *F*(1,114) = 2.28, *p* = .13, *η*_*p*_^*2*^ = 0.02), but they could visualize the positive image less well than the control image (*M* = 6.8, *SD* = 1.7 and *M* = 7.9, *SD* = 1.5, respectively, *F*(1, 114) = 12.60, *p* = .001, *η*_*p*_^*2*^ = 0.10).

### 3.2. Primary outcome: Physical sensitivity

#### 3.2.1. Intensity scores

**Fig 1:**
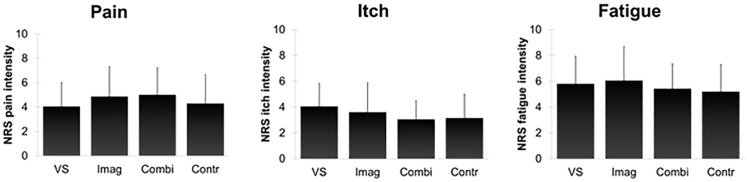
Means and standard deviations of NRS intensity scores for pain, itch, and fatigue during the respective tests. VS = Verbal suggestion condition; Imag = Imagery condition; Combi = Combination condition; Contr = Control condition. Error bars represent standard deviations.

Table 1 and Fig 1 display the NRS intensity scores for pain, itch, and fatigue during the respective tests. The composite intensity score (i.e., the standardized sum score of mean pain, itch, and fatigue intensity during the respective tests indicating physical sensitivity) was not affected by verbal suggestion, imagery, or the combination of both, as indicated by a 2x2 ANOVA (*F*(1,112) = 0.03, *p* = .87, *η*_*p*_^*2*^ < 0.01; *F*(1,112) = 0.49, *p* = .49, *η*_*p*_^*2*^ < 0.01; *F*(1,112) = 1.94, *p* = .17, *η*_*p*_^*2*^ = 0.02, respectively). Age was the only variable that differed significantly between the conditions and that was associated with the composite intensity score, but including age as a covariate did not affect the results.

**Table 1:** Means and standard deviations of NRS intensity and unpleasantness scores of pain, itch, and fatigue during the respective tests.

Condition/Sensation	Verbal suggestion	Imagery	Combination	Control
	(n = 30)	(n = 29)	(n = 28)	(n = 29)
**Pain intensity**	4.1 *±* 1.9	4.9 *±* 2.4	5.0 *±* 2.2	4.3 *±* 2.4
**Itch intensity**	4.1 *±* 1.8	3.6 *±* 2.3	3.0 *±* 1.4	3.1 *±* 1.8
**Fatigue intensity**	5.8 *±* 2.1	6.1 *±* 2.6	5.4 *±* 1.9	5.2 *±* 2.1
**Pain unpleasantness**	4.6 *±* 2.3	5.3 *±* 2.8	5.7 *±* 2.4	4.8 *±* 2.7
**Itch unpleasantness**	3.3 *±* 2.0	3.2 *±* 2.5	2.8 *±* 1.7	2.6 *±* 1.7
**Fatigue unpleasantness**	4.1 *±* 2.5	3.9 *±* 3.1	3.6 *±* 2.3	3.8 *±* 2.5

Exploratory ANOVAs for the separate physical sensitivity tests, conducted to enhance the comprehension of the results for the composite intensity score, indicated that verbal suggestion and imagery did not affect pain, itch, or fatigue (all *p* > .05). There was an interaction effect on itch (*F*(1,112) = 4.57, *p* = .04, *η*_*p*_^*2*^ = 0.04), participants in the *Combination condition* reported less itch than participants in the *Verbal suggestion condition* (*F*(1,56) = 5.71, *p* = .02, *η*_*p*_^*2*^ = 0.09), but there were no interaction effects on pain and fatigue (all *p* > .05).

Post hoc sensitivity analyses indicated that other methods of handling missing data (i.e., not replacing the values, excluding all data from participants with missing values, or replacing missing values with the last observation heightened with the group difference between the missing and preceding value) yielded comparable results. Further post hoc sensitivity analyses provided no evidence of order or time effects: 1) frequency analyses showed that the majority of participants reported no or hardly any remaining or spontaneous pain, itch, or fatigue prior to a subsequent test (≥ 95% NRS scores ≤ 2); 2) univariate repeated measures ANOVAs indicated that pain, itch, and fatigue intensities prior to each test did not significantly differ from or where lower than baseline levels; 3) separate 2x2 ANOVAs regarding pain, itch, or fatigue during only the first, second, or third test yielded the same conclusions as the primary analyses; and 4) including the order of the physical sensitivity tests as a covariate did not affect the results. Furthermore, post hoc sensitivity analyses showed that including baseline pain, itch, and fatigue levels as covariates did also not affect the results.

To determine whether the null results should be interpreted as evidence for the absence of an effect of the expectation inductions, we reanalyzed our data within a Bayesian framework [68, 69]. We calculated the Bayes factor (BF_A0_) using the JAPS software package, in which default priors are used (the null hypothesis is compared to the alternative hypothesis that the effects may occur in either direction) [70–72]. A BF_A0_ smaller than 0.33 is commonly considered to indicate substantial evidence for the null hypothesis, a BF_A0_ larger than 3 is considered to indicate evidence for the alternative hypothesis, whereas a Bayes factor between 0.33 and 3 indicates merely anecdotal or inconclusive evidence for either hypothesis [68,69]. The Bayes factors for the effects of verbal suggestion and imagery on physical sensitivity (BF_A0_ = 0.20 and BF_A0_ = 0.24, respectively) indicated that there was substantial evidence for the absence of an effect of the expectation inductions on physical sensitivity.

#### 3.2.2. Unpleasantness scores

Table 1 displays the NRS unpleasantness scores for pain, itch, and fatigue during the respective tests. The composite unpleasantness score (i.e., the standardized sum score of mean pain, itch, and fatigue unpleasantness during the respective tests) was also not affected by verbal suggestion, imagery, or the combination of both, as indicated by a 2x2 ANOVA (*F*(1,112) = 0.10, *p* = .75, *η*_*p*_^*2*^ < 0.01; *F*(1,112) = 0.47, *p* = .50, *η*_*p*_^*2*^ < 0.01; *F*(1,112) = 0.49, *p* = .49, *η*_*p*_^*2*^ < 0.01, respectively). Exploratory ANOVAs for the separate physical sensitivity tests indicated that verbal suggestion, imagery, or the combination of both did not affect pain, itch, or fatigue unpleasantness (all *p* > .05).

### 3.3. Secondary outcome: Physiological responses

Table 2 displays heart rate and skin conductance at baseline and during the pain, itch, and fatigue tests. Heart rate during the pain and itch tests was not affected by verbal suggestion, imagery, or the combination of both, as indicated by 2x2 ANCOVAs (all *p* > .05). The results were similar after exclusion of the data of three participants with irregular heartbeats (detected during visual inspection of the ECG signals). Skin conductance during the pain, itch, and fatigue tests was also not affected by verbal suggestion, imagery, or the combination, as indicated by 2x2 ANCOVAs (all *p* > .05). The results were similar after the exclusion of the data of one participant who had a very high skin conductance (*z* > 3.29). Non-significant Pearson correlation coefficients were found between the NRS intensity scores for pain, itch, and fatigue during the respective tests and concurrent heart rate and skin conductance (all *p* > .05).

**Table 2:** Means and standard deviations of heart rate and skin conductance at baseline and during the pain, itch, and fatigue tests.

Condition/Time	Verbal suggestion	Imagery	Combination	Control
	(n = 30)	(n = 29)	(n = 28)	(n = 29)
**Heart rate** ^a^ ^,^ ^b^				
**Baseline**	70.5 *±* 10.6	67.5 *±* 9.6	67.2 *±* 9.2	67.8 *±* 9.1
**Pain test**	72.5 *±* 10.8	71.9 *±* 11.5	69.6 *±* 11.2	72.6 *±* 11.7
**Itch test**	68.6 *±* 10.1	67.1 *±* 11.3	65.6 *±* 9.8	68.6 *±* 11.5
**Skin conductance**				
**Baseline**	1.9 *±* 1.8	2.0 *±* 2.1	2.0 *±* 1.3	2.9 *±* 3.2
**Pain test**	5.6 *±* 3.6	5.7 *±* 3.2	6.0 *±* 2.9	7.3 *±* 6.0
**Itch test**	5.4 *±* 3.3	5.1 *±* 2.9	5.6 *±* 2.7	7.2 *±* 5.4
**Fatigue test**	6.2 *±* 3.3	5.6 *±* 2.6 ^c^	6.2 *±* 2.0	7.8 *±* 5.6

^a^ Heart rate during the fatigue test is not reported here because it was tailored during this test

^b^ Full heart rate data missing for 1 participant due to technical problems (*Imagery condition*)

^c^ Skin conductance data fatigue test missing for 1 participant due to technical problems (*Imagery condition*).

### 3.4. Secondary outcome: Psychological characteristics

Positive affect (PANAS PA) and optimism (LOT-R) were not influenced by verbal suggestion, imagery, or their combination, as indicated by 2x2 ANCOVAs (all *p* > .05). Participants only reported less anxiety (STAI-S) after control imagery than after positive imagery (*M* = 25.7, *SD* = 5.5 and *M* = 27.0, *SD* = 6.7, respectively, *F*(1,111) = 4.38, *p* = .04, *η*_*p*_^*2*^ = 0.04), but anxiety was not influenced by verbal suggestion or the combination (all *p* > .05).

None of the psychological characteristics (e.g., neuroticism, imagery ability) moderated the effects of the expectation inductions on physical sensitivity, as indicated by non-significant beta-coefficients for all interactions of the psychological characteristics with verbal suggestion, imagery, or verbal suggestion x imagery (all *p* > .05).

## Discussion

The current study investigated, for the first time, the individual and combined effects of positive verbal suggestion and imagery on physical sensitivity, as indicated by sensitivity to pain, itch, and fatigue. Although both positive verbal suggestion and imagery induced positive expectations, these expectation inductions did not affect physical sensitivity (neither pain, nor itch, nor fatigue), or concurrently measured heart rate and skin conductance.

The finding that the verbal suggestion of reduced physical sensitivity due to a (placebo) capsule did not affect physical sensitivity is in contrast with a substantial body of research that showed that verbal suggestion of the effects of a placebo treatment can effectively reduce pain [6,7,11,12,14]. Other research has also provided preliminary indications that verbal suggestion can reduce itch and fatigue [30–32,34]. However, there are several other studies that could also not confirm the effects of verbal suggestion on pain [10] and fatigue [33]. An important distinction between the current study and previous research is that in our study the verbal suggestion addressed physical sensitivity, encompassing multiple sensations simultaneously, whereas in the majority of other studies verbal suggestion addressed just one sensation. The current findings might thus indicate that generic suggestions are less effective than specific suggestions, although this needs further research. Another important difference concerns the distinction between the experimental and control conditions. Generally, the suggestion that a drug is potent is contrasted with the suggestion that a drug is ineffective [7,10,11,14,32,33] or with no treatment [9], whereas we used a more subtle comparison, between effectiveness in the majority or minority of users. Even though participants expected the capsule to be more effective after positive verbal suggestion than after control verbal suggestion, with a large effect size, indicating that the verbal suggestions were distinguishable, these instructions did not affect sensitivity to physical sensations. However, a similar verbal suggestion regarding the relief of pain or itch for the majority of participants was effective in an earlier study by our group [31]. Thus, especially the specificity of suggestions might be an important predictor of their effectiveness. In future research, this can be further assessed by comparing, for example, instructions addressing physical sensitivity with instructions addressing a single sensation, either alone or in combination with another procedure.

Positive imagery generated more positive and less negative general expectations than control imagery of a typical day, with a small to moderate effect size, but it did not affect physical sensitivity. The original, more general, best possible self (BPS) imagery, however, has previously been found to reduce pain sensitivity and medical care utilization [17,20], although a more recent study using BPS imagery could not replicate the effects on pain [21]. Our adjustment of BPS imagery to enhance specificity and applicability to physical health might have resulted in imagery that was too abstract for participants, possibly because health is often conceptualized in negative terms (e.g., absence of symptoms). Indeed, the participants indicated that they could visualize their best possible health less well than a typical day. Additionally, we found that imagery of health did not increase positive affect, in contrast to BPS imagery [17–19,21], possibly because health is generally only considered when one does not feel healthy and health consequently has a somewhat negative, rather than just a positive, connotation. More specific and concrete images of a desired and positively valued outcome, e.g., imagining diminished pain when a painful hand is bathed in analgesic fluid, might be more effective [73,74]. In addition, it is important to note that participants were not told of the intended effects of the imagery exercise and thus might not have recognized the imagery exercise as an intervention. Although this design allowed us to assess the effects of imagery per se, combining imagery with information about the purpose of imagery (i.e., verbal suggestions) might be essential to its effectiveness. Indeed, neither psychological nor medical treatments are commonly provided without a treatment rationale.

The effectiveness of the combination of positive verbal suggestion and imagery was also explored in the present study. Such a combination is also found in hypnosis [75], which has been found to be able to reduce pain [76,77]. In addition, the combination of verbal suggestion with a more implicit learning procedure, conditioning, has often been found to have larger effects on physical sensitivity than either expectation induction alone [14,30,78]. Our negative finding might partially be explained by the degree of integration of the two expectation inductions; since we were also interested in their separate effects, the capsule and imagery exercise were presented as two different interventions in the current study. This procedure might have been insufficient to generate an additive effect and might even have reduced or counteracted the effects of each individual method. A more effective integration might be achieved by imagery of a suggested treatment outcome or by providing suggestions about the effectiveness of imagery itself.

Lastly, it is important to note a few limitations of this study. First, the assessment of the sensitivity to induced pain, itch, and fatigue in one study allowed us to assess the generic effects of expectation inductions on physical sensitivity, but it might have caused order or time effects. For example, it is known that pain can inhibit itch, that analgesia can induce itch [28,79,80], and that physical exercise can reduce pain [81,82]. However, such interactions are not likely to have affected the results because the physical sensitivity tests were presented in random order with standardized 14-minute intervals between tests [45,83,84]. Additionally, to prevent time effects, participants were reminded about the expectation inductions before each test. Sensitivity analyses provided no evidence of order or time effects: 1) participants’ pain, itch, and fatigue were adequately diminished after the between tests intervals, 2) participants reported equally low or lower pain, itch, and fatigue prior to each of the tests as compared to baseline pain, itch, and fatigue levels, 3) analyses of pain, itch, or fatigue during only the first, second, or third test, yielded the same conclusions as the primary analyses, and 4) statistically controlling for order did not yield differential results for the primary analyses. Second, since we used a sample consisting of healthy participants who were relative young and mostly female, the generalizability of our findings to patients is limited. Third, due to the use of different measures to assess expectations, specifically a numerical rating scale for verbal suggestion and the questionnaire for Future Expectations for imagery, the effects of verbal suggestion and imagery on expectations cannot be directly compared. In future research comparable measures of expectations that are closely related to the contents of the intervention are recommended. Fourth, the possible moderating role of the psychological characteristics (e.g., neuroticism, imagery ability) could only be explored [85]. Future research with larger sample sizes is required to further investigate which psychological characteristics predict the effectiveness of expectation inductions.

In conclusion, the results provide more insight into the essential characteristics of different expectation inductions for reducing physical sensitivity, such as sensitivity to pain, itch, and fatigue, although the limitations should be kept in mind. Our finding that relatively general verbal suggestions and imagery did not affect physical sensitivity, to neither pain, nor itch, nor fatigue, in contrast to previous research, suggests that the level of specificity and concreteness of expectation inductions might be crucial for the applicability of expectation inductions to the treatment of physical symptoms.

## Supporting Information

S1 Text CONSORT checklist. (DOC)

S2 Text CONSORT flow diagram. (DOC)

S3 Text Study protocol. (PDF)
