# Supplementary material for: Inducing Expectations for Health: Effects of Verbal Suggestion and Imagery on Pain, Itch, and Fatigue as Indicators of Physical Sensitivity
Source: PLoS One. 2015 Oct 8;10(10):e0139563. doi: 10.1371/journal.pone.0139563 (PMC4598027; doi:10.1371/journal.pone.0139563)
Supplement: S2 Text — (DOC) [file pone.0139563.s002.doc]

**
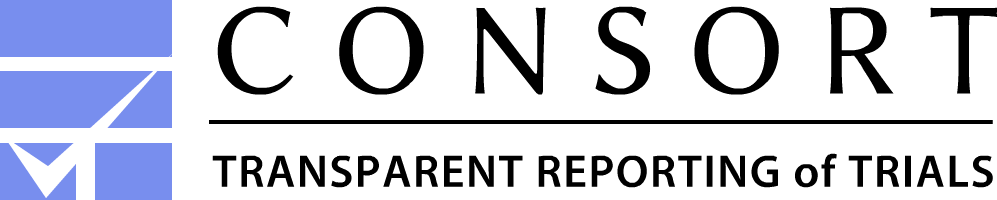
**

**CONSORT 2010 Flow Diagram**

Randomized: n = 116

Peerdeman, van Laarhoven, Donders, Hopman, Peters, and Evers

**Allocation**

**Primary analysis**

**Enrollment**

Registered for participation: n = 195

Excluded: n = 79

♦  Not meeting inclusion criteria: n = 33

♦  Declined to participate: n = 35

♦  Other reasons: n = 11

Allocated to Verbal Suggestion Condition: n = 30

♦ Received allocated intervention: n = 30

Allocated to Imagery condition: n = 29

♦ Received allocated intervention: n = 29

Allocated to Combination condition: n = 28

♦ Received allocated intervention: n = 28

Allocated to Control condition: n = 29

♦ Received allocated intervention: n = 29

Analysed: n = 30

Analysed: n = 29

Analysed: n = 28

Analysed: n = 29
