# Supplementary material for: Inducing Expectations for Health: Effects of Verbal Suggestion and Imagery on Pain, Itch, and Fatigue as Indicators of Physical Sensitivity
Source: PLoS One. 2015 Oct 8;10(10):e0139563. doi: 10.1371/journal.pone.0139563 (PMC4598027; doi:10.1371/journal.pone.0139563)
Supplement: S3 Text — (PDF) [file pone.0139563.s003.pdf]

RESEARCH PROTOCOL

(Version 8, 26 - 09 - 2013)

PROTOCOL TITLE

"Training positive expectations for health: Verbal suggestions versus imagery"

**Protocol ID**	
**Short title**	**Training positive expectations for health**
**Version**	**08**
**Date**	**26-09-2013**
**Coordinating investigator/project leader**	**Prof. Dr. A.W.M. Evers**<br>**Radboud University Nijmegen Medical Centre**<br>**Nijmegen, the Netherlands**<br>**Department of Medical Psychology 840**<br>**Postbus 9101, 6500 HB Nijmegen**<br>**024-3613608**
**Principal investigator**	**Prof. Dr. A.W.M. Evers**
**Sponsor (in Dutch: verrichter/opdrachtgever)**	**Radboud University Nijmegen Medical Centre**<br>**Nijmegen, the Netherlands**
**Independent physician(s)**	**Mw. Dr. M.M.B. Seyger**<br>**Radboud University Nijmegen Medical Centre**<br>**Nijmegen, the Netherlands**<br>**Department of Dermatology 370**<br>**Postbus 9101, 6500 HB Nijmegen**<br>**024-3617239**

PROTOCOL SIGNATURE SHEET

Name	Signature	Date
**Head of Department:**<br><br>**Prof. Dr. J.B. Prins**<br>**Head Department Medical Psychology**		26-09-2013
**Coordinating Investigator/Project leader/Principal Investigator:**<br><br>**Prof. Dr. A.W.M. Evers**<br>**Department of Medical Psychology**		26-09-2013

TABLE OF CONTENTS

SUMMARY 5
1. INTRODUCTION AND RATIONALE 7
2. OBJECTIVES 9
3. STUDY DESIGN 10
4. STUDY POPULATION 13
4.1 Population (base) 13
4.2 Inclusion criteria 13
4.3 Exclusion criteria 13
4.4 Sample size calculation 13
5. TREATMENT OF SUBJECTS 14
5.1 Investigational product/treatment 14
5.2 Use of co-intervention (if applicable) 14
5.3 Escape medication (if applicable) 14
6. INVESTIGATIONAL MEDICINAL PRODUCT 15
7. METHODS 15
7.1 Study parameters/endpoints 15
7.1.1 Main study parameter/endpoint 15
7.1.2 Secondary study parameters/endpoints (if applicable) 15
7.1.3 Other study parameters (if applicable) 15
7.2 Randomisation, blinding and treatment allocation 15
7.3 Study procedures 16
7.4 Withdrawal of individual subjects 19
7.4.1 Specific criteria for withdrawal (if applicable) 19
7.5 Replacement of individual subjects after withdrawal 19
7.6 Follow-up of subjects withdrawn from treatment 19
7.7 Premature termination of the study 19
8. SAFETY REPORTING 19
8.1 Section 10 WMO event 19
8.2 Adverse and serious adverse events 19
8.2.1 Suspected unexpected serious adverse reactions (SUSAR) 19
8.2.2 Annual safety report 19
8.3 Follow-up of adverse events 20
8.4 Data Safety Monitoring Board (DSMB) 20
9. STATISTICAL ANALYSIS 20
9.1 Descriptive statistics 20
9.2 Univariate analyses 20
9.3 Multivariate analyses 21
9.4 Interim analysis (if applicable) 21
10. ETHICAL CONSIDERATIONS 21
10.1 Regulation statement 21
10.2 Recruitment and consent 21
10.3 Objection by minors or incapacitated subjects (if applicable) 21
10.4 Benefits and risks assessment, group relatedness 21
10.5 Compensation for injury 22
10.6 Incentives 22
11. ADMINISTRATIVE ASPECTS AND PUBLICATION 22
11.1 Handling and storage of data and documents 22
11.2 Amendments 22
11.3 Annual progress report 22
11.4 End of study report 23
11.5 Public disclosure and publication policy 23
12. REFERENCES 23

SUMMARY

Rationale: The potential relevance of expectations with regard to treatment outcome for the effectiveness of medical interventions has only sparsely been attended to. This is surprising, given that expectation effects can strongly influence, for example, experienced pain (e.g., Price et al., 1999). Eliciting or enhancing positive expectations might thus be a promising pathway for treating physical symptoms with enhanced effectiveness. Previous research provides evidence that inducing positive outcome expectations of symptom relief, for example pain relief, via verbal suggestions could be a successful method to reduce physical symptoms, such as pain (e.g., Vase et al., 2002; Van Laarhoven et al., 2011). However, these effects have not been found consistently (e.g., Colloca et al., 2008). Alternatively, one can enhance general positive expectations with imagery, as in the Best Possible Self exercise (King, 2001), in which participants write about and imagine their best possible future self. This exercise proved to be successful in increasing positive future expectations (Peters et al., 2010) and reducing sensitivity to pain (Hanssen et al., in preparation). However, the effects of both verbal suggestions and the imagery exercise on multiple physical symptoms simultaneously have not yet been studied, nor have the effects of these methods been compared.

Objective: Our primary objective is to examine the effects of two expectation manipulations (verbal suggestions with regard to an inert substance and an imagery exercise) on self-reported sensitivity to physical sensations of pain, itch, and fatigue. Additionally, the effects on autonomic and endocrine responses and the role of individual characteristics will be explored, including the possible influence of the 5-HTTLPR genotype and other genetic variants on the effects of expectation manipulations, such as verbal suggestions. The results may contribute to the development of innovative therapeutic methods that are intended to enhance the effectiveness of various medical treatments for physical symptoms via altering positive outcome expectations.

Study design: Expectations are induced by verbal suggestions regarding an inert substance (positive versus neutral) and/or an imagery exercise (positive versus neutral). Using a 2x2 factorial design, participants are randomly allocated to 1 of 4 conditions. Participants in condition 1 will receive positive verbal suggestions regarding the substance, after which they will do a neutral imagery exercise (Positive Verbal Suggestions Condition). Participants in condition 2 will receive neutral verbal suggestions regarding the substance, after which they will do a positive imagery exercise (Positive Imagery Condition). Participants in condition 3 will receive both positive verbal suggestions and they will do the positive imagery exercise (Combination Condition). Participants in condition 4 will receive neutral verbal suggestions, after which they do the neutral imagery exercise (Control Condition). Sensations of pain, itch,

and fatigue will subsequently be induced with stimuli of short duration that previously have been validated by the research group and that are safe and not burdensome (cold pressor test to induce pain, histamine iontophoresis to induce itch, and Åstrand cycle test to induce fatigue). Participants will indicate the intensity and unpleasantness of the experienced sensations on visual analogue scales. The autonomic heart rate response, skin conductance response, the salivary autonomic alpha amylase response, and the salivary endocrine cortisol response will be assessed noninvasively.

Study population: Healthy human volunteers (*n* = 116).

Intervention: Expectation effects are induced by means of (1) positive versus neutral verbal suggestions (that it is effective for 95% versus 5% of people) of the effectiveness of a substance that is, unknown to the participants, inert, and (2) a positive versus neutral imagery exercise in which participants focus on either their best possible health in the future or on a typical day.

Main study parameters/endpoints: The primary endpoint of this study is the effectiveness of the expectation manipulations in affecting sensitivity to pain, itch, and fatigue, i.e. the difference between the conditions in sensitivity to pain, itch, and fatigue after the expectation manipulations, as rated on Visual Analogue Scales (VAS).

Nature and extent of the burden and risks associated with participation, benefit and group relatedness: Participants will complete a series of validated questionnaires via internet to assess relevant individual characteristics (30 minutes). Participants will then visit the Department of Medical Psychology of the Radboud University Nijmegen Medical Center once for approximately three hours. The verbal suggestions and imagination exercise will be given. The substance used is inert, i.e., it does not contain any active ingredients. The stimuli used to induce pain, itch, and fatigue have been validated by the research group, are safe and not burdensome. The autonomic and endocrine responses are assessed noninvasively. No risks are involved with participation in this study.

1. INTRODUCTION AND RATIONALE

The role of expectations in medical treatment has been a topic of interest in scientific and clinical communities for many years. Expectations of pain levels have been found to account for a large part of the variance in post-treatment pain ratings (Den Boer et al., 2006; Montgomery & Kirsch, 1997; Price et al., 1999). And expectations of, for example, pain relief after the administration of a pharmacologically inert substance have been found to predict reductions of pain sensations (e.g., De Pascalis et al., 2002). The expectation effect is generally viewed as a considerable “confounder” of aimed therapeutic effects. Consequently, its potential relevance for therapeutic interventions has only sparsely been attended to. However, given the powerful effect of expectations on, for example, experienced pain, eliciting and enhancing positive expectations is a promising pathway for establishing treatments with enhanced effectiveness in reducing physical symptoms. The current study thus focuses on the therapeutic application of expectation effects, by assessing two potentially effective methods to enhance positive expectations that reduce sensitivity to physical sensations.

The first method is to enhance specific positive outcome expectations. A straightforward method to do so, is by giving verbal suggestions regarding the effectiveness of an inert substance, e.g., by informing participants that they are receiving a powerful painkiller. Earlier research provides preliminary evidence that inducing positive outcome expectations of pain relief via verbal suggestions could be a successful method to elicit positive outcomes (e.g., Benedetti et al., 2003; Vase et al., 2002). A verbal suggestion for pain relief has on occasion even been found to increase placebo analgesia to a magnitude that matches that of an active agent (Vase et al., 2003). However, these effects have not been found consistently; for example, Colloca et al. (2008) did not find verbal suggestions to be effective in reducing pain (in contrast to a classical conditioning procedure). Furthermore, although a considerable amount of research has focused on expectation effects in relation to pain, many other physical sensations, such as itch and fatigue, remain relatively unattended to in the examination of expectation effects. A previous study from our research group provides preliminary evidence that itch might be more sensitive to verbal suggestions than pain (Van Laarhoven et al., 2011), but more research is required to support these findings. Additionally, the effectiveness of verbal suggestions in addressing multiple physical symptoms simultaneously has not yet been investigated.

The second method that can be used to apply expectation effects therapeutically is to enhance general positive expectations via imagery. King (2001) developed the Best Possible Self (BPS) exercise, in which participants write about their best possible future self. King found that participants who wrote about their best possible future self for 20 minutes during 4

consecutive days consequently felt less upset, happier and got sick less often than controls who wrote about a typical day. Interestingly, Peters et al. (2010) found that this exercise, enriched with 5 minutes of mental imagery, is also successful in making participants more optimistic; participants reported more general positive expectations of the future (replicated by Meevissen et al., 2011). Further research showed that the BPS exercise was also effective in reducing sensitivity to pain induced with a cold pressor test (Hanssen et al., in preparation). However, the effectiveness of positive imagery in addressing multiple physical symptoms, such as itch, pain, and fatigue, simultaneously has not yet been investigated. Adjusting the BPS exercise so that it focuses more specifically on physical health is likely to enhance the effectiveness of this exercise for a broader spectrum of physical sensations. Thus, we developed a variant of the BPS exercise in which participants are instructed to write and imagine about their best possible health in the future, a Best Possible Health (BPH) exercise.

When assessing the effectiveness of these two manipulations, it is important to assess not only subjective but also physiological responses. Physical sensations of pain, itch, and fatigue are thought to have a broad common psychophysiological basis and to evoke partly corresponding autonomic and endocrine responses. For example, greater low-frequency heart rate variability has been found to be associated with lower ratings of pain during a cold pressor task (Appelhans & Luecken, 2008). And daily stressors have been found to influence the severity of itch in patients with psoriasis by affecting cortisol levels at moments of high stress (Evers et al., 2011). Additionally, animal and, incidentally, human studies show that expectation learning might have direct effects on the autonomic and endocrine system (e.g., Pacheco-Lopez et al., 2006; Price et al., 2008). For example, Pollo et al. (2003) found that placebo analgesia in experimental ischemic arm pain was accompanied by a reduced heart rate. Also, a conditioning procedure has been found to be capable of producing secretive responses of cortisol as well as a growth hormone (Benedetti et al., 2003). Although in this last study direct verbal suggestions of hormone secretion changes were not found to be effective, it remains relevant to assess whether the expectation manipulations studied in the current study can influence the endocrine system. Thus, we will exploratively study the effects of the expectation manipulations on the autonomic and endocrine system, by continuously assessing heart rate, and by assessing salivary alpha amylase and salivary cortisol after both expectations manipulations and after each outcome measure.

Next to potential physiological effects, individual differences in general outcome expectations, imagery skills and attentional focus on bodily sensations are of importance for the understanding of expectation effects. For example, Geers et al. (2007) found that as people were more optimistic (i.e., held more general positive expectations), response to a positive expectation increased and consequently pain experiences reduced (see also Geers

et al., 2010). Furthermore, a general tendency to focus attention on bodily sensations has been found to be associated with higher levels of experienced itch and pain (Van Laarhoven et al., 2010). Also, suggestibility, which includes imagery skills, has been found to affect expectation responses (De Pascalis et al., 2002). Another factor that affects individual differences is genotype. One relevant gene variant is the serotonin transporter length polymorphic region (5-HTTLPR), for which two alleles have been identified, the short (s) and the long (l) allele. This polymorphism has a high frequency in the human population, as approximately 19% is homozygous for the s-allele, 32% is homozygous for the l-allele, and 49% is heterozygous (Lesch et al., 1996). There is accumulating evidence that the 5-HTTLPR-s allele is associated with determinants of expectation, such as an increased Pavlovian conditioning (e.g. Klucken et al., 2012; Lonsdorf et al., 2009), increased sensitivity to phrased descriptions in a decision making task (Roiser et al., 2009), and scores on a creative imagination task (Volf et al., 2009). Therefore, it can be expected that 5-HTTLPR s-allele carriers will show increased responsivity to expectation manipulations, such as verbal suggestions. Similarly, additional genetic variants will be tested for their involvement in these phenotypes.

The effectiveness of verbal suggestions and positive imagery in addressing multiple physical symptoms, such as pain, itch, and fatigue, simultaneously has not yet been investigated, nor have the methods been compared. In the current project, we thus aim to study the effectiveness of both 1) positive verbal suggestions regarding an inert substance and 2) positive imagery of a positive future health in reducing the sensitivity to the physical sensations of pain, itch, and fatigue. In addition we explore autonomic and endocrine responses to the manipulations, as well as the role of individual characteristics, including the possible influence of the 5-HTTLPR genotype and other genetic variants on the effects of expectation manipulations, such as verbal suggestions. The results of this research may contribute to the development of innovative therapeutic methods that are intended to enhance the effectiveness of various medical treatments for physical symptoms via enhancing positive outcome expectations.

2. OBJECTIVES

Primary objective

Our primary objective is to examine the effects of two expectation manipulations (1. verbal suggestions regarding an inert substance and 2. imagining a positive future health) on self-reported sensitivity to the physical sensations of pain, itch, and fatigue.

Primary hypothesis

Based on previous research, it is hypothesized that both (1) positive verbal suggestions regarding an inert substance as compared to neutral suggestions, and (2) the imagery exercise as compared to a neutral exercise result in a lower sensitivity to physical sensations (pain, itch, and fatigue). In addition, it is explored whether the combination of both verbal suggestions and the imagery exercise results in lower sensitivity to physical sensations (pain, itch, and fatigue) than each manipulation individually.

Secondary objectives

Our secondary objective is to explore expectation effects on autonomic (heart rate and alpha amylase) and endocrine (cortisol) responses. Additionally, we will assess the influence of individual characteristics (e.g., dispositional optimism and the 5-HTTLPR genotype and other genetic variants) on the effectiveness of the expectation manipulations.

3. STUDY DESIGN

Expectation effects are induced by verbal suggestions (positive versus neutral) and/or an imagery exercise (positive versus neutral). Using a 2x2 factorial design, participants are randomly allocated to 1 of 4 conditions, which differ only in the contents of the suggestions and imagery. The effects on sensitivity to pain, itch, and fatigue will be assessed by means of the induction of sensations of pain, itch, and fatigue with stimuli of short duration, which previously have been validated by the research group and which are safe and not burdensome (cold pressor test to induce pain, histamine iontophoresis to induce itch, and Åstrand cycle test to induce fatigue). Physiological data of the autonomic heart rate response, skin conductance response, the salivary autonomic alpha amylase response, and the salivary endocrine cortisol response will be assessed noninvasively. The possible role of genotype in candidate genes on the effects of expectation manipulations on itch, pain and fatigue will be investigated by genotyping or sequencing DNA isolated from saliva samples.

Experimental conditions

Condition 1 (Positive verbal suggestions): Participants will receive positive verbal suggestions regarding a substance that is, unknown to the participants, inert. Subsequently, they will perform a neutral imagery exercise.

Condition 2 (Positive imagery): Participants will receive neutral verbal suggestions regarding the substance. Subsequently they will perform a positive imagery exercise.

Condition 3 (Both positive verbal suggestions and imagery): Participants will receive the same positive verbal suggestions as in condition 1, after which they perform the same

positive imagery exercise as in condition 2.

Condition 4 (Control condition): Participants will receive neutral verbal suggestions, as in condition 3. They will additionally do a neutral imagery exercise, as in condition 1.

The conditions are summarized in Table 1.

Table 1

Brief Overview of Conditions

Condition	Verbal suggestions	Imagery exercise
1	Positive	Neutral
2	Neutral	Positive
3	Positive	Positive
4	Neutral	Neutral

Procedure

Participants will be recruited via advertisements (e.g., on the website of the Radboud University Nijmegen Medical Centre). Before registration, they receive an information letter with elaborate information regarding the study (see sections E1 and E3 of the Research File). Additionally, they receive the questionnaires that are made available online via a secured website (RadQuest), or, if preferred by the participant, paper versions will be sent by regular mail. Participants are instructed to fill out the questionnaires prior to their appointment for the testing session. Filling out the questionnaires will take approximately 30 minutes.

The testing session will take place at the Department of Medical Psychology of the Radboud University Nijmegen Medical Centre and will take approximately three hours. After a brief welcome and explanation of the procedures, participants will be asked to sign the informed consent form. After consent, baseline heart rate, skin conductance, alpha amylase, and cortisol assessments will be made. Heart rate and skin conductance will then be continuously assessed during the experimental session. Subsequently, the physical exercise task will be practiced. After a short break, participants will be provided with either positive or neutral verbal suggestions (depending on the condition they were allocated to). All participants then consume the inert substance, after which the imagery exercise will be explained and performed (content depending on the condition, either positive or neutral). Both manipulations will be followed by alpha amylase and cortisol assessments and brief questionnaires to assess changes in expectations. Following the manipulations, the outcome measurements (sensitivity to pain, itch, and fatigue) will be performed in a randomized order,

followed by alpha amylase and cortisol assessments and with breaks of 10 minutes and a short reminder of the manipulations in between. Participants are asked to spit in a special tube to collect saliva for DNA isolation. The experiment will be concluded with several exit questions and a verbal debriefing by the experimenter. See Table 2 for an overview of the full procedures.

Table 2

Overview Procedure

At home	
Recruitment	Advertisement & Information letter
Pre-test questionnaires	Validated questionnaires of individual characteristics, via internet
In lab	
Preparations	Welcome & explanation procedure
	Baseline assessments physiological measures
	Practice physical exercise test
Manipulations	Verbal suggestion (positive or neutral), with substance intake
	Imagery exercise (positive or neutral)
	Physiological and psychological assessments
Outcome measurements*	Pain sensitivity
	Itch sensitivity
	Fatigue sensitivity
	Physiological and psychological assessments
	DNA genotyping or sequencing
Rounding up	Exit questions
	Debriefing
Total duration	Approximately 3,5 hours

* the order of assessment will be randomized as in a cross-over design, with 10-15 minute breaks between the different assessments

4. STUDY POPULATION

4.1 Population (base)

Healthy human volunteers. Participants will be recruited via advertisements (for example on the website of the Radboud University Nijmegen Medical Centre, Nijmegen).

4.2 Inclusion criteria

- Age ≥ 18 years
- Fluent in Dutch language

4.3 Exclusion criteria

- Severe physical or psychological morbidity (e.g., heart and lung diseases, or DSM-IV psychiatric disorders) that would adversely affect participation
- Chronic pain, itch, or fatigue complaints at present or in the past
- instable asthma or allergic rhinitis
- Use of beta-blockers or other medications that influence heart rate, use of pacemaker
- Inadequate health for physical exercise as indicated by positive responses on the PAR-Q (Physical Activity Readiness Questionnaire)
- Raynaud's disease
- Pregnancy
- Participants are required not to consume coffee, tea, cola, energy drinks, or a heavy meal within a 1 hour period prior to the experiment. Nor should they have smoked in the last two hours or, if possible, have used pain killers or sleep-inducing medication and alcohol or other drugs in the last 24 hours. Additionally they are required to refrain from heavy physical exercise 24 hours prior to the experimental session.

4.4 Sample size calculation

After consultation of a statistician (R. Donders), we calculated the required sample size based on the effect sizes found in prior related research that investigated either the effects of verbal suggestions regarding an inert substance or of the imagery exercise on pain. Vase et al. (2002) found an average effect size of d = 0.85 (range -0.64 – 2.29; r = 0.39) for fourteen studies assessing the effects of verbal suggestions on placebo effects on pain, as measured on numeric rating scales and visual analogue scales. Hanssen et al. (in preparation) found an effect size of *partial* η^2 = 0.073 (r = 0.27) when comparing the effect of positive imagery (M = 55.82, SD = 23.52) with neutral imagery (M = 66.27, SD = 17.56) on experienced pain intensity during a cold pressor task, as assessed on a visual analogue scale ranging from 0 – 100.

Taking the lowest effect size into account and an estimated loss of 10% of the data due

to missing values (e.g., due to apparatus not functioning properly), we plan to use a total sample size of 116 participants, with 29 participants per group (i.e., equal allocation to all conditions). This is based on a G*power analysis for the analysis testing the main hypothesis (a two-way ANOVA testing main and interaction effects), with an alpha of 0.05, a desired power of 0.80, an effect size of *partial* η^2 = 0.073, which resulted in an estimated total sample size of 102 participants.

With regard to the genotype analyses, significant effects of the 5-HTTLPR genotype on determinants of expectation were found in studies with comparable sample sizes, for example by Roiser and colleagues (2009).

5. TREATMENT OF SUBJECTS

5.1 Investigational product/treatment

Expectation effects are induced by means of (1) verbal suggestions regarding the effects of an inert substance (positive versus neutral), and (2) imagery (positive versus neutral).

Verbal suggestions: Participants will be told that they will consume a substance that is developed to make one less sensitive to physical sensations such as pain, itch, and fatigue through its effect on processes in the central nervous system. Participants will then be told that in previous research the substance was found to be either effective for 95% of the consumers (i.e., positive verbal suggestions) or for only 5% of the consumers (i.e., neutral verbal suggestions). The substance participants will receive is an inert pill containing inactive ingredients. It will be developed in consultation with the pharmacy of the Radboud University Nijmegen Medical Centre. Participants are not informed that the substance does not contain any active ingredients.

Imagery exercise: Participants will write about either their best possible health (i.e., positive imagery) or a typical day (i.e., neutral imagery) for 15 minutes, after which they will imagine it as vividly as possible for 5 minutes.

5.2 Use of co-intervention (if applicable)

Not applicable

5.3 Escape medication (if applicable)

Not applicable

6. INVESTIGATIONAL MEDICINAL PRODUCT

Not applicable

7. METHODS

7.1 Study parameters/endpoints

7.1.1 Main study parameter/endpoint

The primary endpoint of this study is the effectiveness of the expectation manipulations in affecting sensitivity to pain, itch, and fatigue, i.e. the difference between the expectation conditions with the control condition in sensitivity to pain, itch, and fatigue, as rated on Visual Analogue Scales (VAS). Based on previous research, it is hypothesized that both (1) positive verbal suggestions regarding an inert substance as compared to neutral suggestions, and (2) the imagery exercise as compared to a neutral exercise result in a lower sensitivity to physical sensations (pain, itch, and fatigue) . In addition, it is explored whether the combination of both verbal suggestions and the imagery exercise results in lower sensitivity to physical sensations (pain, itch, and fatigue) than each manipulation individually.

7.1.2 Secondary study parameters/endpoints (if applicable)

The secondary endpoints of this study are to explore the effects of the expectation manipulations on autonomic (heart rate, alpha amylase) and endocrine responses (cortisol), as well as to explore the influence of several individual characteristics on the effectiveness of the expectation manipulations. More specifically, the influence of general outcome expectations, imagery skills, attentional focus on bodily sensations, and current physiological and psychological symptoms will be assessed, as well as the possible influence of the 5-HTTLPR genotype and other candidate genotypes on the effects of expectation manipulations.

7.1.3 Other study parameters (if applicable)

Not applicable.

7.2 Randomisation, blinding and treatment allocation

Participants will be randomly allocated to 1 of 4 conditions. Participants will be kept unaware of the allocation and their condition. The experimenter cannot be kept blind for the conditions due to the clear differences between the instructions provided during the expectation manipulations. The data are scored and analysed by a researcher blind to the

conditions. No indications for breaking the randomization code are indicated.

7.3 Study procedures

Primary outcome assessments

Sensitivity to pain will be assessed with a cold pressor test that was previously validated by our group (e.g., Van Laarhoven, et al., 2010). Participants will be instructed to place their dominant hand in a tank of water at about 4°C for 1 min. The level of pain will be rated at 20, 40 and 60 seconds after immersion on a visual analogue scale (VAS) ranging from 0 (*no pain*) to 10 (*the worst pain imaginable*).

Sensitivity to itch will be assessed with a histamine iontophoresis procedure that was previously validated by our group (e.g., Van Laarhoven et al., 2011). Histamine will be applied by iontophoresis (Chattanooga Group, Hixson, TN, USA). Histamine dihydrochloride (0.5%) will be dissolved in a gel of 2% methylcellulose in distilled water and 2.5 ml will be placed in an electrode, which will be applied to the dominant forearm, 2 cm distal to the lateral epicondyle of the humerus. The reference electrode will be applied to the skin on the lateral side of the triceps brachial muscle. Current level will be set at 0.4 mA and histamine will be delivered for 2.5 min. During histamine application, participants will rate the intensity of itch every 30 seconds on a VAS ranging from 0 (*no itch*) to 10 (*the worst itch imaginable*).

Sensitivity to fatigue will be assessed with a variant of the Åstrand cycling test (Åstrand & Rhyming, 1954). This is a well validated sub-maximal test in which participants will cycle on a fixed exercise bike for 4 minutes at 60-80 revolutions per minute, at an individualized target heart rate. The individualized target heart rate will be calculated by using the Karvonen formula: intensity x heart rate reserve + resting heart rate (e.g., Cheevers & Pettersen, 2007). Where heart rate reserve is equal to the estimated maximal heart rate (220 – age) minus the resting heart rate. The intensity level will be set between 60% and 70% of the heart rate reserve, which is within the range at which people are generally advised to train (60-80%). Workload (watts) required to attain and retain the target heart rate will be individually determined in a practice test of 5 minutes prior to the test itself. Experienced fatigue will be rated every minute on a VAS ranging from 0 (*no fatigue*) to 10 (*the worst fatigue imaginable).*

Autonomic and endocrine responses

Heart rate will be measured continuously during the full experiment as a measure of autonomic functioning, using a MP150 system and AcqKnowledge software (Biopac Systems Inc., Goleta, California; provided with CE marking). During the cycle test, heart rate will additionally be assessed with a standard chest strap (see e.g., Terbizan, et al., 2002).

Skin conductance will be measured continuously during the full experiment as a measure of autonomic functioning, using a MP150 system and AcqKnowledge software (Biopac Systems Inc., Goleta, California; provided with CE marking).

Alpha amylase will be assessed as a measure of autonomic activity, as has been previously done by our group (De Brouwer et al., 2011), after both manipulations and every outcome measurement. Saliva samples will be collected with salivettes (Sarstedt, Rommelsdorf, Germany) and stored at -35 ºC until further biochemical analyses at the department of Laboratory Health.

Cortisol will be assessed as a measure of endocrine activity, as has been previously done by our group (De Brouwer et al., 2011), after both manipulations and every outcome measurement. Saliva samples will be collected with salivettes (Sarstedt, Rommelsdorf, Germany) and stored at -35 ºC until further biochemical analyses at the department of Laboratory Health.

Genotype

DNA will be isolated from the saliva samples collected in Oragene tubes and 5-HTTLPR (variable number tandem repeat (VNTR)) genotyping will be conducted by PCR and subsequent analysis of product lengths, as we performed previously (e.g., Homberg et al., 2008). Additional genotypes will be tested on the longer term, by either classical genotyping or next generation sequencing techniques.

Self-report questionnaires

Several well-validated questionnaires will be administered to assess the influence of individual characteristics that have previously been found to affect expectation effects or sensitivity to pain, itch, or fatigue. More specifically, the influence of general outcome expectations (LOT, FEX, EPQ, adjusted PCS), imagery skills (QMI), attentional focus on bodily sensations (BVS, adjusted PVAQ), medication attitude (BMQ) and current physiological and psychological symptoms (IPAQ, HADS, PANAS, STAI-T) will be

assessed. See Table 3 for an overview and more information. See Section F1 of the Research file for the full questionnaires. Participants will fill out the questionnaires online or with paper and pencil.

Table 3

Overview Questionnaires

Concept	***Questionnaire***	***Citation***
Demographics	Demographic questionnaire	e.g., Van Laarhoven et al., 2011
General outcome expectations	LOT-R (Life Orientation Test)	Scheier et al.,1994
	FEX (Future Expectancies)	Hanssen et al., in preparation
	EPQ-RSS (Eysenck Personality Questionnaire - Revised): neuroticism & extraversion	Eysenck & Eysenck, 1991
	PCS (Pain Catastrophizing Scale), adjusted for physical sensations	Sullivan et al., 1995
Imagery skills	QMI (Sheehan–Betts Quality of Mental Imagery Scale)	Sheehan, 1967
Attentional focus on bodily symptoms	BVS (Body Vigilance Scale)	Schmidt et al., 1997
	PVAQ (Pain Vigilance and Awareness Questionnaire), adjusted for physical sensations	McCracken, 1997
Medication Attitude	BMQ (Beliefs about Medication Questionnaire)	Horne, Weinman, & Hankins, 1999
Physiological and psychological symptoms	IPAQ (International Physical Activity Questionnaire)	Craig et al., 2003
	HADS (hospital anxiety and depression scale)	Zigmond & Snaith, 1983
	PANAS (Positive and Negative Affect Schedule)	Watson et al., 1988
	STAI-T (State-Trait Anxiety Inventory, state version)	Spielberger, Gorsuch, Lushene, Vagg, & Jacobs, 1983

Debriefing

After a few exit questions (e.g., regarding their attention during the imagery exercise) participants will be debriefed. The experimenter will inform each participant orally about the purpose of the study. The participants will be told that the purpose of the study was to assess de influence of expectations on sensitivity to physical sensations. They will be told that we manipulated expectations by differing verbal suggestions regarding the pill and by a differing imagery exercise. Additionally they will be informed that the pill did not contain any active ingredients. Participants will be given the opportunity to ask questions they have regarding the experiment and their participation in it.

7.4 Withdrawal of individual subjects

Participants can leave the study at any time for any reason and without any consequences. The investigator can decide to withdraw a participant from the study for urgent medical reasons.

7.4.1 Specific criteria for withdrawal (if applicable)

Not applicable

7.5 Replacement of individual subjects after withdrawal

Participants who have withdrawn from the study will be replaced by a randomly selected additional participant.

7.6 Follow-up of subjects withdrawn from treatment

Not applicable

7.7 Premature termination of the study

Not applicable

8. SAFETY REPORTING

8.1 Section 10 WMO event

In accordance to section 10, subsection 1, of the WMO, the investigator will inform the participants and the reviewing accredited METC if anything occurs, on the basis of which it appears that the disadvantages of participation may be significantly greater than was foreseen in the research proposal. The study will be suspended pending further review by the accredited METC, except insofar as suspension would jeopardise the participants' health. The investigator will take care that all participants are kept informed.

8.2 Adverse and serious adverse events

Not applicable

8.2.1 Suspected unexpected serious adverse reactions (SUSAR)

Not applicable

8.2.2 Annual safety report

Not applicable

8.3 Follow-up of adverse events

Not applicable

8.4 Data Safety Monitoring Board (DSMB)

Not applicable

9. STATISTICAL ANALYSIS

9.1 Descriptive statistics

Descriptive statistics (means, standard deviations, etc.) of relevant variables will be calculated.

9.2 Univariate analyses

Before conducting each statistical test, its assumptions will be checked (e.g., of normality), and transformations will be made if necessary. For all analyses a significance level of α = 0.05 will be used.

Fristly, between group differences on pre-manipulation variables are checked. In case significant differences appear on possibly confounding variables (age, sex; VAS baseline pain, itch, fatigue; STAI-S, PANAS, LOT-R, FEX), these variables will be controlled for in the main and secondary analyses.

The main hypotheses, regarding the effectiveness of the expectation manipulations in lowering sensitivity to physical sensations (pain, itch, and fatigue) will be tested with a 2 (positive versus neutral verbal suggestions) x 2 (positive versus neutral imagery) ANOVA, with as dependent variable the combined standardized sum score of the VAS ratings of pain (average of ratings during cold water immersion), itch (average of ratings during histamine application), and fatigue (average of ratings during physical exercise). Both main and interaction effects will be examined. Similar ANOVAs will be conducted to analyse the effects of the expectation manipulations on the individual VAS ratings of pain, itch, and fatigue.

The effects of the manipulations on heart rate, skin conductance, alpha amylase, and cortisol will be assessed with 2 (verbal suggestions) x 2 (imagery exercise) ANCOVAs, with as dependent variables heart rate and skin conductance during the three tests, and cortisol and alpha-amylase after the tests, and with as covariate the resting measurements of the respective variables. Both main and interaction effects will be examined.

The association between subjective and physiological responses to the pain, itch, and

fatigue tests will be assessed with correlation analyses.

The influence of individual characteristics (including genotype) will be exploratively tested with regression analyses. Predictors in the analyses will be the individual characteristics, positive versus neutral verbal suggestions (dummy coded), positive versus neutral imagery (dummy coded), the interaction of both expectation manipulations, and the interactions of each individual characteristic with each individual manipulation and the interaction of both manipulations.

9.3 Multivariate analyses

Not applicable

9.4 Interim analysis (if applicable)

In case interim analyses will be done, they will be performed according to the method of Peto, with an alpha of < 0.001 indicating statistical significance (Peto et al., 1976).

10. ETHICAL CONSIDERATIONS

10.1 Regulation statement

The study will be conducted according to the principles of the Declaration of Helsinki (21.10.2008) and in accordance with the Medical Research Involving Human Subjects Act (WMO).

10.2 Recruitment and consent

Healthy participants will be recruited with advertisements (for example advertisements placed on the website of the Radboud University Nijmegen Medical Centre). Before registration, they will receive adequate written information regarding the study, additionally the questionnaires will be provided. Participants will be given one week to consider their decision to participate in the study, during which they can contact the researchers. If participants indeed wish to participate, they will sign the informed consent form at the beginning of the experimental session. See Section E of the Research File for the appropriate documents.

10.3 Objection by minors or incapacitated subjects (if applicable)

Not applicable

10.4 Benefits and risks assessment, group relatedness

No risks are involved with participation in this study. The only burden for participants is investment of time. No direct benefits are expected to be experienced by the participants.

10.5 Compensation for injury

The sponsor has an insurance which is in accordance with the legal requirements in the Netherlands (Article 7 WMO and the Measure regarding Compulsory Insurance for Clinical Research in Humans of 23th June 2003). This insurance provides cover for damage to research subjects through injury or death caused by the study.

1. € 450.000,-- (i.e. four hundred and fifty thousand Euro) for death or injury for each subject who participates in the Research;
2. € 3.500.000,-- (i.e. three million five hundred thousand Euro) for death or injury for all subjects who participate in the Research;
3. € 5.000.000,-- (i.e. five million Euro) for the total damage incurred by the organisation for all damage disclosed by scientific research for the Sponsor as 'verrichter' in the meaning of said Act in each year of insurance coverage.

The insurance applies to the damage that becomes apparent during the study or within 4 years after the end of the study.

10.6 Incentives

Participants will receive a small monetary reimbursement (€40,-) and will be compensated for travelling costs (when the travelling distance is between 15 and 25 km).

11. ADMINISTRATIVE ASPECTS AND PUBLICATION

11.1 Handling and storage of data and documents

Anonymous participant identification codes will be used to link data to participants. The file containing the linking between participant numbers and personal data (e.g., name) will be managed by the researchers and data manager and will be locked for access by others. Collected data (e.g., questionnaires, laboratory results, informed consents) will be stored for a period of 15 years.

11.2 Amendments

All substantial amendments will be notified to the METC that gave a favourable opinion. Non-substantial amendments will not be notified to the accredited METC and the competent authority, but will be recorded and filed by the sponsor.

11.3 Annual progress report

The investigator will submit a summary of the progress of the trial to the accredited METC once a year. Information will be provided on the date of inclusion of the first participant,

numbers of participants included and numbers of participants that have completed the trial, serious adverse events/ serious adverse reactions, other problems, and amendments.

11.4 End of study report

The investigator will notify the accredited METC of the end of the study within a period of 8 weeks. The end of the study is defined as the last patient's last visit. In case the study is ended prematurely, the investigator will notify the accredited METC, including the reasons for the premature termination.

Within one year after the end of the study, the investigator/sponsor will submit a final study report with the results of the study, including any publications/abstracts of the study, to the accredited METC.

11.5 Public disclosure and publication policy

In accordance with the CCMO statement on publication policy, the results of this study will be disclosed unreservedly, i.e., regardless of confirmation or disconfirmation of the hypotheses. The results will be submitted for publication in peer-reviewed journals.

12. REFERENCES

Appelhans, B. M., & Luecken, L. J. (2008). Heart rate variability and pain: associations of two interrelated homeostatic processes. *Biol Psychol, 77*, 174-182. doi: 10.1016/j.biopsycho.2007.10.004

Appelhans, B. M., & Luecken, L. J. (2008). Heart rate variability and pain: associations of two interrelated homeostatic processes. *Biological Psychology, 77*, 174-182. doi: 10.1016/j.biopsycho.2007.10.004

Åstrand, P.-O., & Ryhming, I. (1954). A nomogram for calculation of aerobic capacity (physical fitness) from pulse rate during submaximal work. *Journal of Applied Physiology, 7*, 218-221.

Benedetti, F., Pollo, A., Lopiano, L., Lanotte, M., Vighetti, S., & Rainero, I. (2003). Conscious expectation and unconscious conditioning in analgesic, motor, and hormonal placebo/nocebo responses. *Journal of Neuroscience, 23*, 4315-4323.

Cheevers, A., & Pettersen, C. (2007). Åstrand Bike Test: 6 minute submaximal exercise test. *Skills lab Hogeschool van Amsterdam.* Retrieved from http://scriptiesonline.bib.hva.nl/document/45411

Colloca, L., Sigaudo, M., & Benedetti, F. (2008). The role of learning in nocebo and placebo effects. *Pain, 136*, 211-218. doi: 10.1016/j.pain.2008.02.006

Craig, C. L., Marshall, A. L., Sjostrom, M., Bauman, A. E., Booth, M. L., Ainsworth, B. E. (2003). International physical activity questionnaire: 12-country reliability and validity. *Medicine and Science in Sports and Exercise, 35*, 1381-1395. doi: 10.1249/01.Mss.0000078924.61453.Fb

De Brouwer, S. J., Kraaimaat, F. W., Sweep, F. C., Donders, R. T., Eijsbouts, A., Van Koulil, S. (2011). Psychophysiological responses to stress after stress management training in patients with rheumatoid arthritis. *PLoS One, 6*, e27432. doi: 10.1371/journal.pone.0027432

De Pascalis, V., Chiaradia, C., & Carotenuto, E. (2002). The contribution of suggestibility and expectation to placebo analgesia phenomenon in an experimental setting. *Pain, 96*, 393-402. doi: 10.1016/S0304-3959(01)00485-7

Den Boer, J. J., Oostendorp, R. A. B., Beems, T., Munneke, M., & Evers, A. W. M. (2006). Continued disability and pain after lumbar disc surgery: The role of cognitive-behavioral factors. *Pain, 123*, 45-52. doi: DOI 10.1016/j.pain.2006.02.008

Evers, A. W. M., Verhoeven, E. W. M., Kraaimaat, F. W., De Jong, E. M. G. J., De Brouwer, S. J. M., Schalkwijk, J. (2010). How stress gets under the skin: Cortisol and stress reactivity in psoriasis. *British Journal of Dermatology, 163*, 986-991. doi: 10.1111/j.1365-2133.2010.09984.x

Geers, A. L., Kosbab, K., Helfer, S. G., Weiland, P. E., & Wellman, J. A. (2007). Further evidence for individual differences in placebo responding: An interactionist perspective. *Journal of Psychosomatic Research, 62*, 563-570. doi: 10.1016/j.jpsychores.2006.12.005

Geers, A. L., Wellman, J. A., Fowler, S. L., Helfer, S. G., & France, C. R. (2010). Dispositional optimism predicts placebo analgesia. *Journal of Pain, 11*, 1165-1171. doi: 10.1016/j.jpain.2010.02.014

Homberg,J.R., van den Bos, R., den Heier .E., Suer, R., Cuppen,E. (2008). Serotonin transporter dosage modulates long-term decision-making in rat and human. *Neuropharmacology* 55, 80-84.

Horne R, Weinman J, Hankins M (1999). The Beliefs about Medicines Questionnaire: The development and evaluation of a new method for assessing the cognitive representation of medication. *Psychology & Health*;14:1–24.

King, L. A. (2001). The health benefits of writing about life goals. *Personality and Social Psychology Bulletin, 27*, 798-807. doi: 10.1177/0146167201277003

Klucken, T., Alexander, N., Schweckendiek, J., Merz, C.J., Kagerer, S., Osinsky, R., Walter, B., Vaitl, D., Hennig, J., Stark, R. (2012) Individual differences in neural correlates of fear conditioning as a function of 5-HTTLPR and stressful life events. *Soc Cogn Affect Neurosci. In press*. 10.1093/scan/nss005

Lesch, K.P., Bengel, D., Heils, A., Sabol, S.Z., Greenberg, B.D., Petri, S., *et al*. (1996): Association of anxiety-related traits with a polymorphism in the serotonin transporter gene regulatory region. *Science* 274, 1527-31.

Lonsdorf, T.B., Weike, A.I., Nikamo, P., Schalling, M., Hamm, A.O., Ohman, A. (2009) Genetic gating of human fear learning and extinction: possible implications for gene-environment interaction in anxiety disorder. *Psychol Sci.* 20(2), 198-206.

McCracken, L. M. (1997). "Attention" to pain in persons with chronic pain: A behavioral approach. *Behavior Therapy, 28*, 271-284. doi: 10.1016/S0005-7894(97)80047-0

Meevissen, Y. M. C., Peters, M. L., & Alberts, H. J. E. M. (2011). Become more optimistic by imagining a best possible self: Effects of a two week intervention. *Journal of Behavior Therapy and Experimental Psychiatry, 42*, 371-378. doi: 10.1016/j.jbtep.2011.02.012

Montgomery, G. H., & Kirsch, I. (1997). Classical conditioning and the placebo effect. *Pain, 72*, 107-113. doi: 10.1016/S0304-3959(97)00016-X

Pacheco-Lopez, G., Engler, H., Niemi, M. B., & Schedlowski, M. (2006). Expectations and associations that heal: Immunomodulatory placebo effects and its neurobiology. *Brain Behavior and Immunity, 20*, 430-446. doi: 10.1016/j.bbi.2006.05.003

Peters, M. L., Flink, I. K., Boersma, K., & Linton, S. J. (2010). Manipulating optimism: Can imagining a best possible self be used to increase positive future expectancies? *Journal of Positive Psychology, 5*, 204-211. doi: 10.1080/17439761003790963

Peto, R., Pike, M. C., Armitage, P., Breslow, N. E., Cox, D. R., Howard, S. V., Mantel, N., McPherson, K., Peto, J., and Smith, P. G. (1976). Design and analysis of randomized clinical trials requiring prolonged observation of each patient. I. Introduction and design. *British Journal of Cancer, 34*, 585-612. doi:10.1038/bjc.1976.220

Pollo, A., Vighetti, S., Rainero, I., & Benedetti, F. (2003). Placebo analgesia and the heart. *Pain, 102*, 125-133. doi: 10.1016/S0304-3959(02)00345-7

Price, D. D., Finniss, D. G., & Benedetti, F. (2008). A comprehensive review of the placebo effect: Recent advances and current thought. *Annual Review of Psychology, 59*, 565-590. doi: 10.1146/annurev.psych.59.113006.095941

Price, D. D., Milling, L. S., Kirsch, I., Duff, A., Montgomery, G. H., & Nicholls, S. S. (1999). An analysis of factors that contribute to the magnitude of placebo analgesia in an experimental paradigm. *Pain, 83*, 147-156. doi: 10.1016/S0304-3959(99)00081-0

Roiser, J.P., de Martino, B., Tan, G.C., Kumaran, D., Seymour, B., Wood, N.W., Dolan, R.J (2009). A genetically mediated bias in decision making driven by failure of amygdala control. *J Neurosci*. 29(18), 5985-91.

Scheier, M. F., Carver, C. S., & Bridges, M. W. (1994). Distinguishing optimism from neuroticism (and trait anxiety, self-mastery, and self-esteem) - A reevaluation of the Life Orientation Test. *Journal of Personality and Social Psychology, 67*, 1063-1078. doi: 10.1037/0022-3514.67.6.1063

Schmidt, N. B., Lerew, D. R., & Trakowski, J. H. (1997). Body vigilance in panic disorder: Evaluating attention to bodily perturbations. *Journal of Consulting and Clinical Psychology, 65*, 214-220.

Sheehan, P. W. (1967). A shortened form of Betts Questionnaire upon Mental Imagery. *Journal of Clinical Psychology, 23*, 386-389. doi: 10.1002/1097-4679(196707)

Spielberger, Gorsuch, Lushene, Vagg, & Jacobs (1983). Manual for the State–Trait Anxiety Inventory (Form Y). Palo Alto, CA: *Consulting Psychologist*.

Sullivan, M. J. L., Bishop, S. R., & Pivik, J. (1995). The Pain Catastrophizing Scale: Development and validation. *Psychological Assessment, 7*, 524-532. doi: 10.1037/1040-3590.7.4.524

Thomas, S., Reading, J., & Shephard, R. J. (1992). Revision of the Physical-Activity Readiness Questionnaire (Par-Q). *Canadian Journal of Sport Sciences-Revue Canadienne Des Sciences Du Sport, 17*, 338-345.

Van Laarhoven, A. I. M., Kraaimaat, F. W., Wilder-Smith, O. H., & Evers, A. W. M. (2010). Role of attentional focus on bodily sensations in sensitivity to itch and pain. *Acta Dermato-Venereologica, 90*, 46-51. doi: 10.2340/00015555-0743

Van Laarhoven, A. I. M., Vogelaar, M. L., Wilder-Smith, O. H., Van Riel, P. L. C. M., Van de Kerkhof, P. C. M., Kraaimaat, F. W. (2011). Induction of nocebo and placebo effects on itch and pain by verbal suggestions. *Pain, 152*, 1486-1494. doi: 10.1016/j.pain.2011.01.043

Vase, L., Riley, J. L., & Price, D. D. (2002). A comparison of placebo effects in clinical analgesic trials versus studies of placebo analgesia. *Pain, 99*, 443-452. doi: 10.1016/S0304-3959(02)00205-1

Vase, L., Robinson, M. E., Verne, G. N., & Price, D. D. (2003). The contributions of suggestion, desire, and expectation to placebo effects in irritable bowel syndrome patients - An empirical investigation. *Pain, 105*, 17-25. doi: 10.1016/S0304-3959(03)00073-3

Volf, N.V., Kulikov, A.V., Bortsov, C.U., & Popova, N.K. (2009). Association of verbal and figural creative achievement with polymorphism in the human serotonin transporter gene. *Neurosci Lett,* 463, 154-157.

Watson, D., Clark, L. A., & Tellegen, A. (1988). Development and Validation of Brief Measures of Positive and Negative Affect - The PANAS Scales. *Journal of Personality and Social Psychology, 54*, 1063-1070. doi: 10.1037/0022-3514.54.6.1063

Zigmond, A. S., & Snaith, R. P. (1983). The Hospital Anxiety and Depression Scale. *Acta Psychiatrica Scandinavica, 67*, 361-370. doi: 10.1111/j.1600-0447.1983.tb09716.x
